# Forms of Aid Provided to Refugees of the 2022 Russia–Ukraine War: The Case of Poland

**DOI:** 10.3390/ijerph19127085

**Published:** 2022-06-09

**Authors:** Elżbieta Ociepa-Kicińska, Małgorzata Gorzałczyńska-Koczkodaj

**Affiliations:** Institute of Spatial Management and Socio-Economic Geography, University of Szczecin, 70-453 Szczecin, Poland; malgorzata.gorzalczynska-koczkodaj@usz.edu.pl

**Keywords:** social welfare for refugees, systemic aid, humanitarian crisis

## Abstract

The Twenty-Fourth of February 2022 marked the beginning one of the greatest humanitarian crisis in Europe. Within the first six days of the war, the number of Ukrainian refugees exceeded 4 million, which is more than twice the total number of incomers who arrived in Europe during the Syrian migration throughout 2015. Most of them found themselves in Poland; thus, an urgent need for ad hoc humanitarian and systemic aid arose. To cope with the situation, a number of changes to the Polish law were introduced so as to provide help to the refugees (mainly women and children) from Ukraine. To systematise the changes, the authors of the study analysed the legal acts that had been created or amended for the purposes of providing aid to the refugees staying in Poland. The research study has shown that, in the first days following the war’s outbreak, the measures of key importance were the grassroot initiatives taken by Polish citizens, but as days went by, systemic aid became indispensable. Moreover, non-standard needs had to be handled due to the fact that the refugees were mainly women and children rather than entire families. In conclusion, the war in Ukraine has shown how important it is to have refugee aid procedures at hand and to have a detailed guidance prepared beforehand.

## 1. Introduction

**Figure 1:**
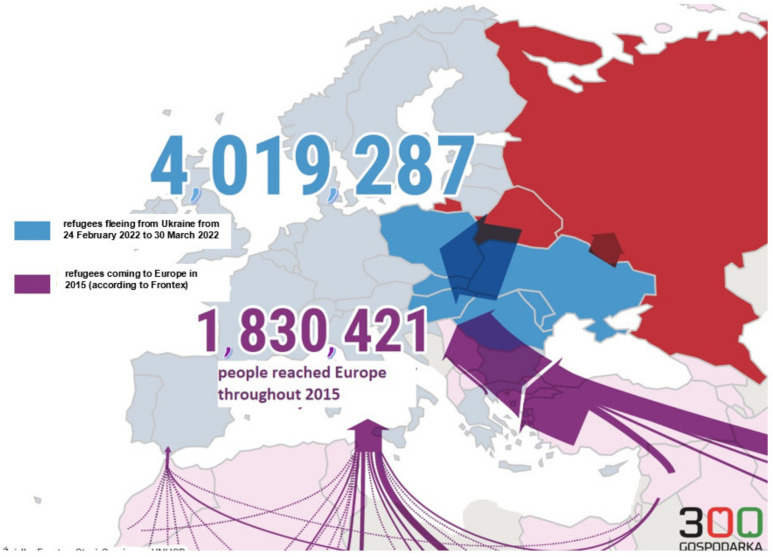
Refugee crises in Europe in 21st century. Source: own study based on 300gospodarka.pl (accessed on 31 March 2022) [3].

The 2022 Russian invasion of Ukraine triggered one of the largest and fastest refugee movements seen in Europe since the end of WWII. It is estimated that by 2 March 2022, within just a week from the war’s onset, 874,000 people sought refuge in neighboring countries. According to the UN data as of 19 March 2022, the number of Ukrainian refugees exceeded 3.3 million [1]. The United Nations High Commissioner for Refugees (UNHCR) anticipated that, in the first weeks of the war, as many as 4 million refugees might flee from Ukraine, whereas the European Union (UE) estimated that the total eventual number might be 7 million. No matter which of the estimates is correct, it is obvious that Europe is facing unprecedented refugee-related challenges [2]. By 19 March 2022, the greatest number of refugees, i.e., 2010.7 thousand, arrived in Poland, 518.3 thousand arrived in Romania, 359.1 thousand arrived in Moldavia, 299.3 thousand arrived in Hungary, 240.0 thousand arrived in Slovakia, 184.6 thousand arrived in Russia, and 2.5 thousand arrived in Belarus. According to the data published at 300gospodarka.pl (accessed on 31 March 2022), on 30 March 2022, the number of Ukrainians escaping abroad to escape from the war exceeded 4 million [3], which is more than twice the total number of incomers who arrived in Europe during the wave of migration throughout 2015 (Figure 1). The number of refugees who crossed the Ukrainian–Polish border on 24 February 2022–11 April 2022 exceeded 2.68 million.

**Figure 2:**
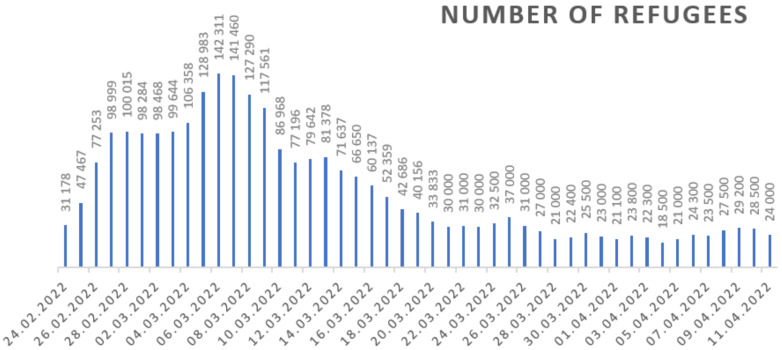
Number of refugees who crossed the Ukrainian–Polish border on 24 February 2022–11 April 2022. Source: own study based on daily data published by the Border Guard [6].

More people come to Poland every day (Figure 2), and these were mainly women and children in whose case it was first necessary to take care of their basic physiological needs (food and sleep). The next step is to provide them with safety and security, defined in the first place as not being affected by poverty or shortage [4] and next as a stable and relatively foreseeable environment in which an individual or a group may pursue their goals without disturbance or harm and without concerns about such disturbance or harm [5].

In accordance with the Maslow’s hierarchy of needs, the need for safety is a basic human need. This way or other, meeting many other life needs is predicated on fulfilling this one safety need [7]. The images of destruction caused by war and the sound of warning horns and exploding bombs all affect the psyche of both adults and children. Since the war’s outbreak, Ukrainian children with their mothers and grandmothers spent days and nights in cellars and then they travelled for dozens of hours to arrive in a foreign country. Lines of vehicles queueing to cross the Ukrainian–Polish border stretched for dozens of kilometers, so people left their cars to reach the border faster on foot. Some of them let go of their luggage where they had packed a tiny part of their life’s possessions and the most essential things because it hindered their run for safety. Eventually, they crossed the border with a small backpack while starved and exhausted. Those travelling on an overcrowded train had to stand squeezed throughout the journey that took even 60–80 h since they left home. In the world of the 21st century, such pictures evoke shock, disbelief, and outrage, but first and foremost, it invokes an urge to help those people. In the wake of the war that broke out nearly 6 weeks before writing this paper, a need arose to provide ad hoc emergency help on an enormous scale to thousands of women and children who in their escape from the war found themselves in Poland. Poles showed enormous involvement in supporting refugees from Ukraine. In view of that exceptional situation, a quick reaction was of key importance. The ad hoc actions taken by citizens of Poland made it possible for the refugees to survive “here and now”; however, as subsequent thousands of refugees had been arriving in Poland day by day, the need for systemic aid was becoming more and more evident.

Studies regarding systemic solutions dedicated to providing support (with particular emphasis on social welfare) to refugees, including children, were authored by, i.a., Aurino and Giunti, International Labour Organization, Makhema, and the UN Refugee Agency [8,9,10,11]. Andrade, Sato, and Hammad [12] analysed and underlined the need to enable refugees to feel that they belong to the community in which they live. In this context, it should be stressed that the recent literature (2015–2021) focuses mainly on migrants/refugees from Syria, and in addition to challenges, it addresses the dilemmas regarding the balance between the protection of refugees and protection of native societies [2,13]. Researchers also analysed the priorities for relocations and their perception, i.e., in terms of educational possibilities, financial stability and social connections, local language command level, friendships and contacts at school, and extent of integration with the local community [13,14]. Experts and politicians’ opinions highlight the gap between the approach to refugees from Syria and refugees from Ukraine. They raise questions about the limits of humanity: For example, what is the difference between a Syrian mother with a child from a Ukrainian one? The subject of differences in attitudes towards refugees and their “segregation” is very complex. The common cultural features and the similar language of both nations should be noted. In addition, since 11 June 2017, there is a visa-free regime between Poland and Ukraine, which resulted in an influx of Ukrainian citizens to work in Poland. Thanks to this, Poles had the opportunity to meet the citizens of Ukraine, accept their presence in their country, and establish relations, friendships and relationships with them. In addition, historical threads, such as World War II, mean that Poles, seeing the attack on Ukraine, are aware of the military threat also to their own country.

In our studies, we focused in the first place on systemic changes and legislative changes prepared on a very short notice and passed in an extraordinary and unprecedented manner in Poland. A review of those changes poses the question whether or not any country should always have such solutions at hand, just in case, or whether we should assume the greatest added value is tailoring them to match a particular situation that could not have been anticipated. We believe that this paper may constitute a good point of reference for international analyses and comparisons, as well as a source of inspiration for other countries which face or will face a similar situation. Due to the extraordinary circumstances, our analyses cannot show the effects of implementation or post factum statistics. We decided to focus on the issue from the perspective of the required quick systemic changes and their assumptions and expected effects in the form of social welfare for refugees. In the next stage, following the end of the war in Ukraine, we are planning to examine the effectiveness of the solutions described herein.

The issue of providing help to refugees affected by war is definitely an interdisciplinary one, as it regards virtually all aspects of life. This article concentrates on the ones connected with the economic and social challenges, first and foremost on systemic support which is necessary in view of the enormous inflow of women and children. As we now have to deal with the largest and quickest movement of refugees faced by Europe since the end of WWII and the first such an enormous movement in the 21st century, in the digital era of fast information flow, we are defining the research niche by reviewing quickly made changes to the national law in order to accommodate it to the situation connected with the inflow of millions of refugees to Poland. The importance of the events is confirmed by the fact that, in March 2022, for the first time in history, the European Commission asked the European Council to state the existence of a massive influx of displaced persons from Ukraine and to introduce temporary protection. Thus, it initiated the launch of protective solutions provided for by Council Directive 2001/55/WE.

The aim of this study is to analyse and systematise the forms of aid dedicated to refugees from Ukraine so as to obtain a set of good practices that could be useful or inspiring for other countries. We focused on Poland due to the fact that it received the largest number of refugees from Ukraine. The study is based on a detailed review of amendments to the Polish law implemented since 24 February 2022. The structure of this paper is as follows: The next part presents the theoretical issues connected with the functioning of refugees in new communities and the broadly understood aspect of social welfare that they should be provided. Next, the methodology is described. The subsequent section presents the outcomes of our analyses that lead to discussion and conclusions presented in the last part of this article.

## 2. Theoretical Background

According to the Geneva Conventions, a refugee is a person who, owing to well-founded fear of being persecuted for reasons of race, religion, nationality, and membership of a particular social group or political opinion, is outside the country of their nationality and is unable or, owing to such fear, is unwilling to avail themselves of the protection of that country or who, not having a nationality and being outside the country of their former habitual residence as a result of such events, is unable or, owing to such fear, is unwilling to return to it [15]. In the European Union, a refugee is defined as a person who has a well-founded fear of being persecuted in their home country for reasons of race, religion, nationality, political opinion, or membership of a particular social group, and their status was acknowledged by the host country [16]. The refugee law and the international law on human rights are closely interconnected, as refugees flee from governments that are unable or unwilling to protect the fundamental human rights. In cases where in the context of an armed conflict there is fear of persecution and compromised safety or risk of death, refugee law also overlaps with the international humanitarian law [17]. At this point, it is important to distinguish between the “refugee” and “migrant” concepts. A migrant can be defined as a person who decides to move to another country to find a job, pursue educational ambitions, join their family, or for other personal reasons rather than because of a direct risk of losing life or freedom. As opposed to refugees, migrants do not fear being persecuted or seriously harmed in their native countries. Migrants still enjoy the protection of their native state governments even abroad, and they can return home [12].

Over the past decade, the world has been facing dilemmas related to refugees whose numbers almost doubled in the years 2010–2017 (for more details, please refer to [18]). The 2022 Russian invasion of Ukraine has increased the numbers considerably. Research studies that describe this issue feature debates regarding, i.e., refugees’ humanitarian needs, negative consequences of their mass migration in the context of host country economies [18], or the risk of a cross-border spread of conflicts [19]. Some researchers have voiced an opinion that the economic impact of refugees on the host countries is controversial and not quite understood [20], insufficiently researched, and difficult to be unambiguously evaluated [19]. According to Artooz and co-authors [21], refugees may also have a positive effect on the economy and the society in the host regions, demonstrating local entrepreneurial activity [20,22]. Undoubtedly, the economic impact of refugees is predicated on a number of additional variables, i.a., the host country’s reaction, the level of offered social welfare, and—over a longer time horizon—on the human capital they are contributing.

Having experienced the traumas of war, refugees have to face new stressors connected with being a minority in a foreign country that include learning a foreign language and new social and cultural norms [21], family-related problems (i.e., being separated from the family and conflicts with relatives), and financial or housing problems or unemployment. It was shown that the stressors related to relocation derive from experiences that are detrimental to mental health, leading in particular to post-traumatic stress disorder (PTSD), anxiety, and depression [23]. Therefore, such a challenging task such as helping refugees to fully participate in and to contribute to the society should engage authorities at all levels (central, regional, and local) as well as many other entities. These include real estate agencies, job centres, schools, health care centres, media, NGOs, employers, trade unions, sports clubs, religious institutions, neighbours, classmates, co-workers, and a countless number of other community members. Their contributions, big and small, have an impact on the situation of refugees who lost their communities and the sense of belonging and are starting from scratch in a foreign land [12]. The UN Refugee Agency [24] specifies three major areas of intervention—social welfare, family aid services, and economic integration centres—whereas according to the United Nations International Children’s Emergency Fund, social welfare is defined as all interventions of public, private, and voluntary organisations as well as informal networks in order to support communities, households, and natural persons in their efforts aimed at preventing, managing, and overcoming threats and weaknesses and at improving the social status and rights of marginalised people [10]—this definition will be applied in this study on providing aid to refugees. Social welfare systems cover social networks of aid or social security as well as intervention programmes on the labour market [11]. Social welfare is not permanently available to migrants, refugees, and asylum seekers in most developing countries around the world. Providing access to social welfare is one of the functions of the national legislature and policy, which in turn ensues from relations between the hosting communities and migrants, as well as the willingness and ability of governments as such to promote an open society [13]. The EU Directive laying down standards for the reception of applicants for international protection indicates that member states are required to guarantee the fundamental right to human dignity, which theoretically is binding in all EU countries, but in practice it is executed differently depending on the country [25]. In practice, in 2015/2016, the EU reacted to the influx of refugees from Syria with a phenomenon known as “defensive integration” (as a reaction to the lack of top-down regulations and developed mechanisms to respond to this situation. Under the EU Directive 2001/55 /WE, setting out the rules for granting temporary protection in the event of a mass influx of displaced persons and measures supporting a balance of efforts between EU countries in 2022 the EU introduced temporary protection for people fleeing the war in Ukraine. On this basis, persons who have been a permanent resident in Ukraine and left the country on or after 24 February 2022 to escape the war may be entitled to temporary protection for at least one year in any EU country. There are significant differences between the implementation of long-term, sustainable social welfare programmes and exceptional or extraordinary measures [26]. “Extraordinary” or “humanitarian” aid is predicated on the assumption that the former concentrates on long-term structural issues, whereas humanitarian aid addresses ad hoc, temporary needs ensuing from a crisis [8], concentrates on short-term, one-off actions to save lives and to meet urgent needs of people affected by a crisis, and it is often implemented via external entities and using external sources of funding. However, even though reacting in crisis situations is characterised by focusing on urgent aid to meet the needs in order to save lives, in reality, it is rarely the case. Secondly, although humanitarian aid is characterised by meeting urgent needs, there is evidence showing that when scalable local social welfare systems are in place, they can be put to work faster than humanitarian actions. This was confirmed by Ulrichs and Sabates-Wheeler who pointed to the example of the Hunger Safety Net programme in Kenya, which is capable of providing aid in emergencies within 10 days from declaring a state of emergency. For comparison, it takes 9 months for the United Nations to react [8]. Humanitarian social welfare is aimed at preventing, mitigating, or solving of crises, regardless of whether this is performed by local governments within their boundaries or by international humanitarian organisations.

Despite the existence of global regulations in the form of the international humanitarian law, refugee law, and the law on human rights, which act as complementary legal regulations that share one aim being protection of life, health, and dignity of people [27], in a situation when hundreds of thousands of people are coming into a given country, there is a need for concrete solutions tailored to the actual and current circumstances at local, regional, and national levels. The 2022 Russian invasion of Ukraine has demonstrated the unresolvable challenge concerning Europe’s ability to adopt an international system of refugee protection [2]. Not all countries welcome Ukrainian refugees with open arms. As it could be seen in Poland at the end of February and the beginning of March 2022, in the first days of the war, active citizenship was of key importance. The quickest and the most effective aid was the one organised on an ad hoc basis by individuals, NGOs, business entities, and institutions that normally do not deal with humanitarian aid, such as schools, sports complexes, or dormitories, who/which offered meals and places to sleep to refugees. People quickly set up support groups in social media, which were dedicated to providing help to Ukrainians and aimed at exchanging information, i.a., about the current material needs (clothes, blankets, cleaning agents, food, baby care products, etc.). Those actions were efficient and effective on a “here and now” basis; however, as more and more thousands of refugees poured in, the situation called for long-term systemic aid that would help the refugees to settle down in a foreign country. This is a particularly challenging task due to the fact that women and children prevailed among the refugees (their husbands and fathers are fighting in the war), and one of the characteristics of the refugee children’s life is the state of constant insecurity regarding plans for the future [13].

## 3. Methodology

This study was based on the observation method in connection with the current situation of refugees from Ukraine who arrived in Poland, their needs, and the forms of support they are offered. The document review method was used to analyse the changes in the law that are directly connected with providing aid to Ukrainian citizens that were made after 24 February 2022. The structure of the study makes it possible to systematise and understand the process of providing systemic aid to refugees, its key challenges, and constraints connected with them (using also the case study method). We decided to concentrate on one country only (i.e., Poland) in order to fully account for the social context and institutional environment. Secondly, Poland received the greatest number of refugees from Ukraine, hence its significance. As this is a highly current issue and we are at the early stage of data collection, there are considerable opportunities to discover new aspects in terms of securing refugees’ life and health in this exceptional situation where the refugee population is dominated by women and children.

The analysis covered the major legal acts that directly or indirectly affect the possibility of providing additional forms of aid to Ukrainian refugees arriving in Poland. The obtained information was systematised and tabulated. Moreover, this article strives to provide the most current data provided by the Border Guard as for the number of refugees from Ukraine crossing the border from 24 February 2022 to 11 April 2022, as well as expert opinions (due to the highly current nature of the issue, these are published mainly in the media) regarding the humanitarian crisis, the challenges connected with it, and potential solutions. Additionally, we attempted to identify some grassroot initiatives that were of key importance at the first stage of providing help to refugees.

## 4. Results

According to UNICEF, social welfare is rooted in the international human rights rules and it is the key tool for policies aimed to build up resilience, fight poverty, and improve economic and social outcomes among individuals and families in a difficult situation. The 1951 Refugee Convention specifies, i.e., refugees’ right to work; however, the national regulations that govern the right to work are mediated by the political economy and safety factors, which often results in constraining refugees’ access to the labor market [28]. In Poland, 12 March 2022 was the day of coming into effect of the extraordinary Act on Aid for Ukrainian Citizens in Connection with the Armed Conflict in the Territory of Ukraine [29], binding from 24 February 2022 (hereinafter: the Act on Aid for Ukrainian Citizens).

The Act on Aid for Ukrainian Citizens specifies in detail the principles of legalising the residence of Ukrainian citizens who entered the territory of the Republic of Poland directly from the territory of Ukraine in connection with the acts of war taking place in that country.

The Act also specifies the following:

1. Detailed principles for providing jobs to citizens of Ukraine who have a right of legal residence in the Republic of Poland;
2. Aid provided by voivodeship governors to citizens of Ukraine;
3. Establishing a new special fund in the state budget to finance aid for citizens of Ukraine, in the amount of PLN 3 billion;
4. Some of the rights of Ukrainian citizens with legalised residence in the territory of the Republic of Poland;
5. Detailed principles of extending the periods of legalised residence of Ukrainian citizens and any documents issued to them by Polish authorities with regard to their right to enter and reside in the territory of the Republic of Poland’
6. Some of the rights of the Polish and Ukrainian citizens being college/university students, academic teachers, or research fellows leaving the territory of Ukraine;
7. Detailed regulations regarding education, upbringing, and taking care of children and schoolers being citizens of Ukraine, including support for local administrative units (LAUs) with regard to implementation of additional education tasks in that respect;
8. Detailed principles of organising and functioning of tertiary education institutions in connection with providing Ukrainian citizens with study opportunities.

The Act on Aid for Ukrainian Citizens also stipulates the principles of providing social welfare to Ukrainian citizens. In accordance with the adopted legal regulations, in justified cases, a voivodeship governor may provide aid to such people:

1. Accommodation;
2. Providing collective catering on a full board basis;
3. Providing transport to the place of accommodation between such places or to facilities run by the Head of the Office for Foreigners based on the regulations of the Act on Providing Protection to Foreigners or to places where medical care is provided to Ukrainian citizens.

According to Professor Duszczyk, an expert from the Centre of Migration Research, we are now dealing with a crisis that we have never experienced before. People from Syria or Libya who fled from war mostly ended up in refugee camps where they stayed for years without a possibility of employment, with limited education opportunities, and without a possibility of movement. Those who had managed to enter Europe did not enjoy the rights that Ukrainians now have. The procedure of temporary protection (granted for 18 months in Poland, and for 12 months in other EU countries) makes it possible for refugees to avail themselves of education and medical care, to take up a job, and most importantly, to move freely between the EU countries. These conditions (described in more detail in Table 1) may entice Ukrainians to stay in Poland. One of the most important rights that is viewed as a privilege is the possibility for Ukrainian refugees to obtain a Polish personal identification number (PESEL) which enables them not only to seek employment but also provides access to all kinds of social welfare, healthcare, and education. From the first days of issuing PESEL numbers to Ukrainians, it is possible to observe increased activity on the labour market, which indicates that refugees want to be financially independent and self-sufficient as soon as possible. Moreover, a PESEL number is necessary for anyone who wants to obtain social benefits to which Ukrainian refugees are entitled. In addition to that, pursuant to the provisions of Art. 12 para. 4 of the Act on Aid for Ukrainian Citizens, any local administrative unit (LAU) may, on their own initiative and within the scope of funds being at their disposal, provide aid to Ukrainian citizens. This is a statutory delegation regarding expenditure from the budget of any given LAU. For such aid to be legal and lawful, the engaged LAUs have to adopt appropriate resolutions (the public sector may implement only the tasks and activities which ensue from the legal regulations).

Moreover, a number of facilitations and departures from the existing regulations have been introduced, such as the following:

1. Establishing the Aid Fund aimed at financing or subsidising tasks that are connected with providing help to refugees (established at the BGK Bank, analogically as in the case of the COVID-19 Counteracting Fund). All self-governments are required to keep funds obtained from the Aid Fund in a separate bank account. The persons responsible for appropriation of the funds are the head/mayor of the municipality or the executive boards of poviat or voivodeship (i.e., first- and second-order administrative unit). They are also required to develop a financial plan, to notify of any unused funds and to return them, and also to submit information about the plan implementation. The financial resources coming from the Aid Fund are to substantially contribute to faster and easier implementation of (both ad hoc and systemic) aid tasks.
2. LAUs and their associations may provide help local and regional communities. In the hitherto binding regulations, aid in kind as well as financial aid provided to other LAUs was only possible within the territory of Poland. Very often, e.g., in the wake of a natural disaster, some local administrative units provided help to other LAUs being in need. Currently, this regulation makes it possible to provide aid to communities based in the territory of Ukraine.
3. Waiving the constraints stipulated in the Act on Public Finance [34] (Art. 128 para. 2) and the Act on Revenues of Local Administrative Units [35] (Art. 42 para. 3). The amended legal regulations also waive the upper limit of subsidies that can be provided to LAUs to finance their statutory day-to-day and investment tasks (which previously amounted to max. 80% of the task implementation costs) and the limit of subsidies to finance statutory tasks being investment projects related to schools and educational institutions (which previously amounted to 50%). The amended regulations make it possible for LAUs to obtains funds from the above-mentioned source to the extent greater than before, which will be necessary to implement aid projects.
4. Moreover, the principles of computing the ratio of day-to-day expenses to revenues, specified in Art. 242 of the Act on Public Finance [34], have been softened by excluding any aid expenses from the computation. This modification also pertains to the ratio that constrains a LAU’s debt repayment amount in 2023 and subsequent years (Art. 243).
5. Regardless of failing to meet the requirements stipulated in Art. 29 para. 9–12 of the Act on Public Finance [34], it is possible for the National Rehabilitation Fund for the Disabled (PFRON) to amend its financial plans with the aim to support Ukrainian citizens with disabilities.
6. In order to enable faster reactions to the need to implement aid tasks and to ensure necessary funds to finance them, there is a possibility of authorising the head/mayor of the municipality by the local/regional council of the LAU to perform the following:
  - Make amendments to the revenue and expense plan connected with the LAU’s budget, including transfers between budget classification categories;
  - Make amendments to the long-term financial forecast and to the expense plan connected with the LAU’s budget, in connection with implementing new investments or making investment purchases by the LAU, as long as that change does not deteriorate the LAU’s budget outturn.
7. Amendments have been made in connection with the need to apply the Public Procurement Law [36]. In the case of public procurement carried out in connection with specific kinds of aid for refugees, the provisions of the Act of 11 September 2019 (Public Procurement Law) do not apply at all.
8. It is also possible for, i.a., LAUs to outsource the implementation of a public task to NGOs, other public benefit organisations, and trade unions without the need to announce an open tendering procedure as per the Act on Public Benefit and Voluntary Organisations [37]. It is also possible to outsource implementation of public health tasks without the need to hold a bidding contest. These regulations are aimed at streamlining the prompt outsourcing of tasks related to providing aid to Ukrainian citizens.
9. A certain novelty is the introduction (in connection with implementation of refugee-related tasks) of a possibility for a voivodeship governor to issue mandatory orders to specified entities (such as self-government bodies, self-government legal persons, or self-government organisational units without legal personality). The mandatory orders are issued in the form of immediately enforceable administrative decisions that do not have to be substantiated or even issued in writing (they may be given orally or take the form of a written note, sent by e-mail). The mandatory orders as a rule may not pertain to resolving of any matters settled via an administrative decision, and also, they may not concern any operating, exploratory, or investigating activities or any activities connected with offence prosecution. Tasks covered by mandatory orders fall within the scope of state government administration for which LAUs receive funding, and in case they complete them earlier, they are reimbursed the cost. Prior to the amendment of the regulations, in accordance with court rulings, LAUs were not allowed to use their own funds to pre-finance any ordered tasks falling within the scope of state government administration. Now this practice is allowed.
10. The Minister of Education and Science issued an ordinance that amended the regulations regarding details of public schools and kindergartens organisation [38]. The amended ordinance specifies the number of children in a class, in schools and kindergartens, taking into account additional children from Ukraine who fled to Poland to get away from the war.
11. Moreover, tax relief was introduced for those who helped Ukrainians, which is meant to be some sort of compensation for Polish citizens and companies engaged in providing aid for Ukrainian refugees and victims of war. The tax relief for providing aid to Ukrainians covers both PIT (Personal Income Tax) and CIT (Corporate Income Tax), as well as VAT (Value Added Tax). It mainly provides a possibility of deducting any donations made to Ukrainian war victims in end-of-year PIT and CIT returns [39].
12. Moreover, a zero VAT rate was introduced for goods and services that constitute aid for Ukrainian refugees. However, in 2022, 0% VAT may only be applied by enterprises under certain conditions:
  - The goods and services must be handed over as aid for Ukrainians via specified aid agencies, such as, e.g., LAUs, healthcare centres, or the Material Reserves Agency;
  - The donor should sign an appropriate agreement on goods and services supply with the aid agency (this can also take the form of a written statement which must be signed by both parties).
13. Another tax deduction opportunity is blood donation for Ukraine: In the annual tax return, it is possible to deduct the money equivalent of the blood/serum donated free-of-charge.
14. Classifying donations for Ukrainians as tax deductible costs. This regards not only aid in kind or financial donations but also free-of-charge services provided to refugees, e.g., medical care.

In addition to that, many LAUs started bidding procedures in an urgent mode, under the Act on Public Benefit and Voluntary Organizations [37], providing funds for the implementation of various actions to support Ukrainian refugees. This first and foremost concerns psychological counselling. Moreover, the funding from the general reserve to a large extent has been used to perform ad hoc day-to-day tasks connected with helping people from Ukraine, e.g., purchase of beds and adapting buildings for temporary or permanent occupation by refugees.

As part of systemic and organisational measures, 36 reception points have been established in Poland (as at 31 March 2022) [40]. Reception points are places where refugees may have a rest, eat a meal, avail themselves of medical assistance and psychological counselling, file an application for international aid, fill in documents necessary to obtain the right of residence, obtain a referral to one of refugee centres for Ukrainians, and receive information about possibilities of transport to other Polish cities. The reception points are located in every voivodeship (NUTS2 region), and naturally, most of them are operating in regions neighbouring with Ukraine (Lubelskie and Podkarpackie voivodeships). It is not required for Ukrainians to register at the reception point to enter the territory of Poland, but if any incomers seek a refugee status, they need to file an appropriate application. All vital information can be found at the Polish website in the Ukrainian language [41].

From the perspective of systemic aid, the fact that the refugee population is dominated by women and children generates additional challenges connected with giving children a sense of security and offering their mothers or guardians support in childcare so that they can take up a job and be self-sufficient. According to UNICEF’s estimates of 16 March 2022, since the outbreak of the war, 55 children per minute fled from Ukraine, which means that almost, as every single second passes, a child becomes a refugee. UNICEF also pointed out that children desperately need safety, stability, and protection, especially those who move about without a carer or were separated from their families [42]. By 30 March 2022, the Social Insurance Institution received ca. 145,000 applications for the 500+ child benefit claimed for the children displaced from Ukraine [43]. The “Aktywizacja bez granic” foundation, which operates at the Federation of Polish Entrepreneurs, has taken on new duties consisting in helping to organise afterschool clubs and kindergartens for Ukrainian children. The main idea is that the Ukrainian women who want to engage in paid work must also find a place where their children are taken care of [44]. Some companies that need new workers declared that as a result of the pandemic their employees had switched to teleworking, leaving empty office space that could be converted to a company kindergarten for refugees’ children.

Moreover, any Ukrainian children and youths aged up to 19 who arrived in Poland may be vaccinated against contagious diseases in Polish medical centres (in accordance with the Polish mandatory vaccination scheme). If they stay in Poland for more than three months, vaccinations will be obligatory for them [45]. The Polish Minister of Education and Science has stressed that it is Poland’s duty to take care of the education of Ukrainian refugees’ children and he proposed that the children should go to a Polish school when they are ready, i.e., when they have rested and recuperated from war trauma. Each child of Ukrainian refugees has a right to free education in a Polish school, but before they can be enrolled, they also have a right to connect with their schools in Ukraine [46]. In addition to the aid addressed for children and youths, Polish universities as well as various foundations and associations started free-of-charge courses of Polish for Ukrainian adults. This will help them to function and assimilate better in Poland to find a job and to integrate with society.

Particularly in the first days following the war’s outbreak, when systemic aid was not yet ready and large numbers of refugees started flowing into Poland, it was NGOs that provided a major part of the aid. It was the NGOs that were the first to rise to the occasion rather than wait for legal regulations or to receive funding for the aid. Associations and foundations have been relying on volunteers who have been helping for free. Many organisations managed to obtain aid in kind, i.e., food and many other articles for which they did not need to pay in any stores or to any corporate or private persons or other organisations. We should all appreciate the fact that so many people have been helping refugees with such dedication and selflessness and that volunteers have been waiting at railway stations to welcome Ukrainians and offer them a meal or a soft toy for a child as well as a package of vital organisational information. NGOs as such do not have sufficient financial resources, as they can finance their activities only from membership fees, subsidies received from public funds, donations and aid in kind, sponsors, and predominantly the 1% tax donations that they receive as public benefit organisations. The engagement of NGOs in providing aid to refugees was appreciated also by the Polish-American Freedom Foundation (PAFF), which decided to donate extra PLN 4 million to help Ukraine. “We Support Ukraine” is a new PAFF programme addressed for Polish NGOs that immediately and on a massive scale brought aid to Ukrainians in need. The goal of the Programme is to provide aid to civilians affected by the war in Ukraine via taking actions such as the following [47].

Supporting initiatives taken by Polish non-governmental organisations in cooperation with Ukrainian partners in Ukraine:

1. Supporting initiatives implemented by Polish non-governmental organisations for the benefit of Ukrainian refugees;
2. Supporting coordination of non-governmental organisations’ activities for the benefit of Ukraine (i.e., in cooperation with other institutions/environments)—mainly with regard to refugees from Ukraine, but also initiatives implemented in Ukraine itself;
3. Subsidies may be obtained for initiatives taken by Polish organisations as well as those implemented in cooperation with Ukrainian partners, especially for the following:
  - Intervention projects related to activities aimed at, i.a., ensuring safety to civilians, provision of medical supplies and personal protection devices, equipping and adapting some places to enable functioning during the war, and also activities to support the refugees in Poland (grants amounting to PLN 25,000);
  - Long-term projects covering activities that are aimed, i.a., at supporting the education of children and schoolers in Poland, the adaptation of Ukrainian war refugees to the Polish labour market and counteracting their discrimination (grants amounting to PLN 45,000).

The Polish NGOs, described here in the context of grassroot initiatives, operate under legal regulations such as the Act on Associations [48], the Act on Foundations [49], and the Act on Public Benefit and Voluntary Organisations [37]; thus, they constitute a certain part of the system. However, their operations are voluntary—they may take up initiatives, but they are not required to do so. This can be seen in the estimates made by the Polish Central Statistical Office (GUS) in 2021, according to which out of 138 thousand NGOs, only ca. 70 thousand were actively operating [50].

## 5. Discussion and Conclusions

On 24 February 2022, Russia invaded Ukraine, and the first refugees entered the territory of Poland. Exactly one week later, i.e., on 3 March 2022, the Ministry of Internal Affairs and Administration presented a bill which on 12 March 2022 was adopted by the Polish Parliament (the Act on Aid for Ukrainian Citizens in Connection with the Armed Conflict in the Territory of Ukraine) [31]. Thus, during the first two weeks from the war’s outbreak, there were no extensive, systemic solutions in place, and the aid provided to refugees was initiated ad hoc on a grassroot basis, as it was needed immediately (humanitarian aid). In the first days following the refugees’ arrival, the most urgent and basic needs had to be met, such as having a rest, a hot meal, and a warm place to sleep. However, later on, it was necessary to provide them with systemic aid that would enable them to become self-sufficient and allow them to work, which also involved providing care for the children who arrived from Ukraine. If entire Ukrainian families had come, it could be assumed that men would take up jobs to support their families, while women would take care of their children. However, in this case, there is an armed conflict, so the men stayed in Ukraine to fight, and the refugees are mainly women and children, which means that childcare must be provided so that the mothers can go to work.

The refugee statistics quoted in the Introduction show the inflow of refugees over time and provide grounds to believe that the reception points can only serve the ad hoc, basic needs of the people who arrive in Poland. After that, the facilities must be ready to receive the subsequent waves of refugees. It is also important to note here that thousands of Poles have been picking up refugees straight from the Ukrainian–Polish border and take them to their homes. Soon after the war’s outbreak, some local politicians such as city mayors urged the government to provide systemic solutions and pointed out that no country could cope with such a large humanitarian crisis [51,52,53]. The first refugees who arrived in Poland often had some friends or relatives who were able to take care of them. However, as days went by, there were more and more people coming, and they had nobody in Poland to go to.

The Russian invasion of Ukraine and all its consequences, which we describe in this paper, make us pose a question on whether or not countries should always be prepared for wars or similar events and have some systemic solutions at hand. Analysing this aspect with the example of Poland, it should be stressed that there are specific procedures in place that are broken down into operational tasks to be carried out on the individual self-government levels (local and regional governments) or by specified entities in the public finance sector. The procedures were prepared to be applied in case of armed military actions. However, the situation faced by Poland in the first quarter of 2022 evidently showed a gap in this area—there are no systemic solutions ready to be applied in such circumstances. An important topic, which additionally emphasizes the need for a systemic organization of support for refugees, is their safety. This includes verifying the credibility of people accepting refugees and training volunteers and employees at reception points in detecting signs of a threat to the safety of women and girls, including trafficking human beings or other exploitation methods. According to the Human Rights Watch report [54] the government should immediately develop and implement consistent protocols that ensure protection at reception points, and for all refugee transportation and housing, all refugees should receive clear information about how to mitigate protection risks, seek help, and report incidents.

The war in Ukraine has shown how important it is to have such procedures and a detailed solution package prepared beforehand. The sixteen days that elapsed from the war’s outbreak to coming into effect of the act on providing aid to Ukrainian refugees was a short time for drafting a bill from scratch, but on the other hand, it was a very long period of time from the perspective of refugees needing help “here and now”. The sixteen days’ time was a test for the Polish society when it was immediately necessary for individuals and NGOs to take ad hoc actions to help those in dire need. In view of the lack of systemic solutions (at the moment of the war’s outbreak), without the grassroot initiatives and prompt actions, the refugees would definitely have been in a much worse situation or maybe it would have been impossible for them to obtain help. The provided comprehensive aid and the warm welcome that Ukrainian citizens were given in Poland helped them assimilate faster. In addition to helping the subsequent waves of their compatriots arriving in Poland, as a sign of gratitude, Ukrainian refugees have been engaging in a number of initiatives to benefit local Polish communities, such as tidying up parks or public utility buildings and holding Ukrainian cooking workshops, festivals, and concerts featuring Ukrainian artists.

## Figures and Tables

**Table 1:** Types of aid for which Ukrainian refugees are eligible grouped by task.

Type of Aid	Description	Source of Funding/Operations
Financial Aid		
Establishing/distribution of provisions	A new special fund was established to finance aid for citizens of Ukraine, in the amount of PLN 3 billion. Establishing of the provision will not require an opinion of the Parliamentary Committee responsible for budgeting. The funds from this provision will be used to finance or subsidise tasks in the area of public safety and order protection, national border protection, border and foreigners traffic control, and coordination of activities connected with the state’s migration policy, crisis management, civil defence, fire safety, citizenship and vital records, and national identity cards and passports, including in particular aid provided to citizens of Ukraine by voivodeship governors.	state budget, central government administration
Child benefit (500+)	Refugees may obtain the 500+ child benefit for each child under 18 years of age.	state budget, central government administration (Social Insurance Institution)
“Start of School” benefit	A benefit amounting to PLN 300.00 to help parents to buy school supplies such as backpacks, notebooks and other necessary things.	state budget, central government administration
Family Care Capital	Ukrainians who live with their children in Poland are eligible for this benefit. The amount of this benefit may total PLN 12,000 per child and it is applicable for a child aged from 12 to 35 months on the condition that it is the second or subsequent child of the parents. Eligibility does not depend on the parents’ income. Similarly, as in the case of Polish citizens, the Family Care Capital benefit may be paid out in two ways: PLN 500 paid monthly for 2 years; PLN 1000 paid monthly for 1 year.	state budget, central government administration
Subsidy for crèche	Subsidising the cost of crèche in the maximum amount of PLN 400.00 per month to cover the cost of crèche or children’s club or day-time child minder. Only parents who do not receive the Family Care Capital benefit are eligible for this subsidy.	state budget, self-government administration
Social Welfare—cash benefits and benefits in kind	Ukrainian refugees may apply to be covered by social welfare which will be provided to them without a standard home study. This encompasses various benefits: Cash allowance—these can be regular, periodic, or ad hoc benefits to cover basic necessities of life benefits in kind, e.g., providing temporary accommodation, food, clothes, etc. As a rule, social welfare and benefits are to support individuals and families with the lowest income or without any livelihood, i.e., those threatened by poverty and in an extremely difficult situation.	State budget, self-government administration (municipal welfare centers)
Granting one-off cash allowance	Support in the form of one-off cash allowance intended for covering the living costs, in particular costs of food, clothes, footwear, means of personal hygiene, and accommodation-related costs. The benefit amounts to PLN 500 in the case of a single-person household and PLN 300 per person in cases where there are more people in the household. An application for the benefit should be filed in writing in the municipality where the Ukrainian citizen resides. Granting the benefit does not require issuing an official decision.	state budget, self-government administration (municipality)
Cash allowance for hosting	A municipality may enter into an agreement with “any entity” upon granting a cash benefit for a period of maximum 60 days (which can be extended in particularly justified cases) in return for ensuring accommodation and full board to Ukrainian citizens amounting to PLN 40 per person per day.	state budget, central government and self-government administration (municipality)
Nursing care and healthcare benefits	The benefits depend on the financial situation of applicants: - Family benefit (PLN 95–135 per month); - Attendance benefit (PLN 215.84 per month); - Attendance allowance (PLN 2119 per month); - Carer’s benefit (PLN 620 per month).	state budget, central government administration
Waiving donation tax on aid received by Ukrainians	The tax relief for aid obtained by Ukrainian refugees will make it possible for them to avoid paying personal income tax for all kinds of support they get, i.e., free-of-charge use of accommodation made available by Polish families, free-of-charge catering, medical services, or even language courses and occupational training.	LAU budgets, self-government administration
Applying the crisis provision	In accordance with the interpretation of the Regional Chamber of Audit, self-governments may also use funds from the crisis management provision to cover expenses connected with the refugee crisis, e.g., purchase of food, medications, etc., that can be handed over to specified organisations, directly to refugees arriving in Poland or Ukrainians staying in Ukraine. In addition to that, foreigners may receive individual aid targeted at a specific person in accordance with the Act of 12 December 2013 on foreigners. Before the war outbreak, any foreigners including refugees could not benefit from the crisis management provision funds, as the only applicable aid was possible under the Act on foreigners.	LAU budgets, self-government administration
EU funds	Possibility of providing food in the form of food packages or meals under the Food and Basic Material Assistance Operational Programme 2014–2020 co-financed by the Fund for European Aid to the Most Deprived (FEAD). The assistance is available for the citizens of Ukraine whose residence in the Republic of Poland has been legalised.	EU budget, central government and self-government administration
**Healthcare/psychological counselling**		
Psychological care	Free psychological counselling the psychological counselling to be ensured by head/mayor of the municipality in which any given Ukrainian citizen resides.	state budget, self-government administration (State Fund for Rehabilitation of Disabled Persons)
Medical care	Access to medical care covers healthcare services to the same extent and on the same principles as in the case of any persons covered by obligatory or voluntary health insurance, under the Act on Public Medical Care.	state budget, LAU budgets, healthcare units.
**Access to education**		
Access to higher education institutions	No fees are charged from Ukrainian citizens who are students of public tertiary education institutions. Moreover, they may apply for maintenance grants and student loans.	state budget, public tertiary education institutions
Possibility of obtaining maintenance grants	The education system stipulates providing support to students in the form of social aid. School student grants may also be provided to schoolers being Ukrainian citizens, and they take the form of either total or partial payment for educational classes, or education-related aid in kind. A school benefit is part of social welfare and it is intended for schoolers temporarily experiencing a difficult financial situation.	state budget, LAU budgets, self-government administration, educational establishments
Facilitating employment of teachers and teacher’s assistants in schools	In schools and kindergartens where additional classes and groups have been organised to accommodate children and students from Ukraine, the headmaster may assign teachers (with their consent) overtime amounting to more than 1/2 of their weekly obligatory working time.	state budget, LAU budgets, self-government administration, educational establishments
Employing Ukrainian citizens as teachers based on the Teacher’s Charter Act	Ukrainian citizens may be employed in schools, kindergartens (to conduct hobby classes), and in public psychological and educational counselling centres. In this case, the person employed does not have to meet the qualifications criteria. It is enough for such a person to have appropriate background, which is assessed by the headmaster who may find the background proper even without diploma nostrification.	state budget, LAU budgets, central government and self-government administration
Access to crèche, kindergarten and school	The process of establishing other locations where classes may be taught has been simplified to the maximum, and they may be established throughout the school year without changing the resolution in which the school network was determined. In the school year 2021/2022, the number of children in kindergarten and preschool classes may be increased from the current number of 25 up to 28, and in primary schools, in forms from 1 to 3, the number may be increased from 25 up to 29. An extra subsidy for a Ukrainian child, amounting to ca. PLN 5000. Preparatory classes do not have to be located in school buildings, and State Treasury companies will make available locations for such classes.	state budget, LAU budgets, self-government administration
**Administrative and legal counselling**		
Assigning personal identification numbers (PESEL)	Assigning personal identification numbers (PESEL) to Ukrainian citizens.	state budget, LAU (municipality) budget, self-government administration

Source: own study based on [29,30,31,32,33].

## Data Availability

Not applicable.
